# Radiographic Healing Outcomes of Apical Periodontitis Following Endodontic Therapy: A Retrospective Longitudinal Study in a Romanian Cohort

**DOI:** 10.3390/jfb17060304

**Published:** 2026-06-18

**Authors:** Sorina G. Zahiu, Mircea Riviș, Ciprian Roi, Alexandra Roi, Ovidiu Frățilă

**Affiliations:** 1 Doctoral School of Biomedical Sciences, University of Oradea, 410087 Oradea, Romania; sorina_zahiu@yahoo.com; 2 University Clinic of Anesthesiology and Oral Surgery, Research Center of Dento-Alveolar Surgery, Anesthesia and Sedation in Dental Medicine, “Victor Babeș” University of Medicine and Pharmacy, Eftimie Murgu Square No. 2, 300041 Timisoara, Romania; rivis.mircea@umft.ro; 3 University Clinic Oral Pathology, Multidisciplinary Center for Research, Evaluation, Diagnosis and Therapies in Oral Medicine, “Victor Babes” University of Medicine and Pharmacy, Eftimie Murgu Sq. No. 2, 300041 Timisoara, Romania; 4 Department of Medical Disciplines, Faculty of Medicine and Pharmacy, University of Oradea, 410087 Oradea, Romania; ovidiufr@yahoo.co.uk

**Keywords:** apical periodontitis, PAI score, endodontic treatment

## Abstract

Apical periodontitis is a common inflammatory oral condition and a major cause of endodontic treatment need. The present retrospective clinical study aimed to evaluate the frequency, distribution, and radiographic healing of teeth diagnosed with apical periodontitis following primary endodontic treatment or nonsurgical retreatment within a specific patient cohort. Consecutive patients presenting for endodontic treatment at the study clinic between 2020 and 2021 were screened for inclusion. Eligible cases were those in which patients provided written informed consent, presented with periapical inflammatory pathology, and underwent conservative endodontic treatment. Exclusion criteria were incomplete data, non-functional or non-restorable teeth, third molars, pregnancy, probing depth ≥ 4 mm, radiographic bone loss, pathologic tooth mobility due to attachment loss, periodontal involvement of the lesion, and primary dentition. A total of 277 teeth, all diagnosed with apical periodontitis at baseline, were included. Some patients contributed more than one tooth. All treatments were performed by a single operator according to a standardized clinical protocol, including uniform diagnostic criteria, chemo-mechanical preparation, irrigation regimen, obturation technique, and radiographic follow-up at 12 and 24 months. Periapical healing was assessed radiographically using the Periapical Index (PAI). Within this cohort, elderly patients significantly represented the largest proportion of those treated (*p* < 0.001). Maxillary teeth also comprised a significantly higher proportion of cases than mandibular teeth (55.2% vs. 44.8%). The mean initial PAI score was 3.37 ± 0.9 points, with a median of 3 points, and the final score was 1.31 ± 0.93 points, with a median of 1 point. Radiographic healing was observed in 56.68% of cases at 12 months and in 84.84% of cases at 24 months. Primary endodontic treatment and nonsurgical retreatment of teeth with apical periodontitis in this selected patient population were associated with substantial radiographic improvement over a 24-month follow-up period. These findings support the value of standardized endodontic management and longitudinal radiographic monitoring.

## 1. Introduction

Apical periodontitis is an endodontic infection characterized by inflammation and destruction of the tissues surrounding the tooth root, caused by long-term exposure to irritants from an inflamed or necrotic pulp or a failed endodontic treatment [1]. Bacterial contamination can occur in teeth with or without previous endodontic [2]. Dental caries is a global public health problem, being the most prevalent condition, with approximately 2 million people affected [3].

Apical periodontitis is, after dental caries, the most prevalent oral infection, thus being a frequent pathology in dental practice and a public health problem. In a systematic review and meta-analysis on the global prevalence of apical periodontitis, it was highlighted that this pathology is very common in different communities [4].

Studies in the literature have repeatedly demonstrated an association between periapical health and the quality of endodontic treatments. There are studies that have reported that there is a very large number of incorrectly treated teeth [5,6,7]. This explains why teeth that have endodontic treatments have a higher prevalence of this pathology than teeth without endodontic treatment [8,9,10].

It is known that composite resins used for tooth fillings undergo a shrinkage process after polymerization, of approximately 2–5%, thus making it possible for bacteria and toxins to infiltrate through the space created between the restoration and the tooth [11,12]. The direct effects of composite resins on the dental pulp are uncertain. It is not known whether they can cause irreversible pulp inflammation, necrosis and subsequently apical periodontitis [13].

The treatment of choice for apical periodontitis is non-surgical endodontic treatment. It is necessary to monitor the periapical status through periodic follow-up sessions to see the evolution of treatments [14].

In teeth with pulp necrosis and with apical periodontitis, some studies have shown a healing rate of 86% after endodontic treatment [15]. The estimated healing rate for patients who develop AP after undergoing treatment, in practice, is between 62 and 82% [16,17,18].

A search of the endodontic literature revealed a lack of data about the causes, frequency, and distribution of endodontically and non-endodontically treated teeth with apical periodontitis in a subpopulation from Romania. Also, no studies evaluate the success rate after treatment/retreatment of these endodontic lesions in this area. This investigation assessed the frequency, distribution, and success rate after treatment/retreatment of teeth with apical periodontitis using panoramic and periapical radiography.

## 2. Materials and Methods

### 2.1. Study Population

This is a retrospective study focusing on epidemiological, clinical, and radiological characteristics of apical periodontitis.

Our study was approved by the Ethics Committee of the Faculty of Medicine and Pharmacy Oradea (CEFMF/4/30.10.2023), and patients agreed and signed an informed consent form that followed the guidelines of the Declaration of Helsinki.

A total of 1004 patients presenting for endodontic treatments at the study host clinic between 3 January 2020 and 30 September 2021 were available for the present study. Each subject completed an informed consent form and a detailed anamnesis form. After clinical and radiographic examination, a total of 200 patients, with a total number of 277 teeth with apical periodontitis, required endodontic treatments.

### 2.2. Radiographic Examination and Evolution

In order to assess the number of teeth with apical periodontitis, they were clinically and radiologically examined using panoramic digital radiography. A total of 1004 panoramic radiographs (20,072 teeth) were analyzed at the initial imaging examination. All digital orthopantomographs were taken using the Kavo Orthopantomograph™ OP 3D digital device (KaVo, Tuusula, Finland), made by the same dental professional according to the exposure parameters appropriate for the patient’s gender, age and weight. Subsequently, based on this information, in order to obtain a detailed evaluation of the periapical status, the teeth that were diagnosed with AP were re-evaluated with periapical radiographs, using the parallelization technique (SOPRO Imaging software, Sopro 2.53). All periapical radiographs were taken and interpreted by the same dental professional, in order to ensure homogeneity of results.

The method of viewing the radiographs was standardized: the radiographs were examined using a computer, in a darkened room, where the ambient light was controlled for optimal contrast.

The periapical status of all teeth (except for third molars) was examined using periapical index scoring system (PAI), proposed by Ørstavik et al. which scores the apical area of the radiographic images as follows: 1. normal periapical structures; 2. small changes in the bone structure; 3. changes in bone structure with some mineral loss; 4. periodontitis with a well-defined radiolucent area; 5. severe periodontitis with exacerbating features. A score of PAI 1 and 2 was defined as a normal periapical region, and PAI scores (3, 4, and 5) were evaluated as apical periodontitis. One observer with 6 years of clinical experience in endodontics examined the radiographs. All radiographs were evaluated again to determine intra-examiner agreement concerning the detection of periapical radiolucency, and the Kappa coefficient was applied. In the case of multi-rooted teeth, the biggest root with the biggest PAI score was chosen.

After completing the treatment, each patient was informed about the significance of undergoing long-term follow-up examinations. Patients were advised to notify their attending dentist if they observed any changes in their dental status. Post-treatment evaluation was done at 12 and 24 months intervals by taking periapical radiographs for periapical healing assessment. Each time, patients were evaluated for the presence or absence of signs and symptoms such as pain on palpation/percussion and swelling.

Healing outcomes were defined a priori on the basis of periapical tissue status and were independent of intra-operative procedural events. A tooth was classified as “healed” when the PAI score was 2 or lower, and no clinical signs or symptoms were present at the follow-up examination.

### 2.3. Clinical Examination

Each patient completed the medical questionnaire and underwent a panoramic radiograph, followed by clinical examination. The clinical examination included an interrogation, an inspection, palpation, a cold test, percussion, and periodontal probing. After confirming the diagnosis of apical periodontitis through the clinical examination and the radiological evidence of the lesion, a periapical radiograph of the causal tooth was performed.

The type of apical periodontitis was classified according to the American Association of Endodontists classification (Table 1).

### 2.4. Inclusion and Exclusion Criteria

The inclusion criteria encompassed patients who provided signed informed consent to participate in the study, presented with inflammatory pathology located at the periapical level, and requested and followed conservative curative treatment.

The exclusion criteria were represented by patients with missing data, teeth deemed non-functional or non-restorable, wisdom teeth, pregnant women, teeth with a pocket depth of 4 mm or more, visible bone loss on radiographs, and pathologic tooth mobility due to insertion loss, periodontal involvement in the lesion (periodontal lesion mimicking endodontic lesion or true endo-periodontal lesion), or primary dentition.

### 2.5. Protocol

After dental anesthesia (plexus/block to avoid injection into the area of inflammation), the tooth was isolated with the dental dam. In cases where the teeth showed dental posts, massive caries or composite fillings that were marginally mismatched, it was decided to remove them and, where necessary, completely rebuild the coronal part to obtain an optimal sealing. All teeth were occlusally reduced.

In the per-primam group, the instrumentation process was initiated with #08, #10 or #15K-files (Dentsply Maillefer, Ballaigues, Switzerland) to eliminate possible interferences, then the working length was established with an electronic apex locator (Root ZX, Morita, Japan) connected with the appropriate K-file. Then, Hyflex CM/EDM instruments were driven by a VDW Gold electric motor (VDW, Munich, Germany), operating at a speed of 300 rpm and a torque of 2.5 N.cm. The canals were instrumented and shaped using the crown-down technique.

In the retreatment group, after removing dental post, broken instruments or excessive gutta-percha with woodpecker ultrasonic endo tips (Woodpecker Ultrasonic Scaler U6 Led), instrumentation started with 25/.12 Hyflex EDM, then VDW RECIPROC^®^ R25 was inserted in the canal at the estimated working length. In all this time, the integrated apex locator from VDW.GOLD^®^RECIPROC^®^ was activated. After the preparation, the apical fit was evaluated again.

The same canal irrigation protocol was applied to both the primary and retreatment groups. During instrumentation, canal irrigation was performed with 5.25% NaOCl and for the final irrigation in both groups, with 5 mL of 17% ethylenediaminetetraacetic acid (EDTA), followed by 5 mL of 5.25% NaOCl; the canals were dried with absorbent paper points.

Warm vertical compaction technique was used to fill the root canal system with guttapercha and filling paste (AH Plus, Dentsply, Germany) was introduced into the root canal using master cones.

A total-etch bond (Single bond 2, 3M ESPE, St. Paul, MN, USA) technique was used according to the manufacturer’s instructions prior to coronal restoration. Flowable resin composite (Gc Gradia Direct Flo, GC Corporation, Tokyo, Japan) was used as a base material in order to seal the canal orifices, and the coronal restoration was made with resin composite (Gradia Direct, GC Corporation, Tokyo, Japan). In the cases where a fibre post was used, HiRem quartz fibre post (Over Fibers, Imola, Italy) was cemented with Connexio^®^ Dual-Cure (Centrix Inc., Shelton, CT, USA) and Maxcem Elite™ (Kerr Corporation, Brea, CA, USA) as composite core.

In situations where adequate drying of the root system could not be achieved, calcium hydroxide (Cerkamed, Stalowa Wola, Poland) was used, and the access cavity was sealed with Teflon and glassionomer cement (Kavitan Cem Pentron, Jičín, Czech Republic). Patients were rescheduled, and the definitive endodontic obturation was performed one week later.

Instrumentation differed by design between the primary and retreatment groups to address distinct clinical needs. HyFlex CM/EDM (Coltène/Whaledent AG, Altstätten, Switzerland) was used for primary canal preparation to achieve controlled crown-down shaping with a predictable taper, aligning with contemporary guidelines that emphasize safe, conservative shaping in intact canals. Reciproc Blue (VDW GmbH, Munich, Germany) was selected for retreatment because its reciprocating single-file approach facilitates efficient obturation removal and canal cleanup, reflecting guidance that supports streamlined retreatment workflows when obstructions or prior obturation materials are encountered. The operator’s preference is to use HyFlex for per-primam treatment and Reciproc Blue for retreatment, a choice that was predefined in the protocol and based on canal status and manufacturer guidance. Post-endodontic restorations were completed in the same session by the same dental professional under identical clinical conditions for all treated teeth.

### 2.6. Data Collection

Several categories of variables were followed for each patient as follows.

General variables: gender, age in years, reason for presentation (pain, periodic check-up, pre-prosthetic (esthetics), smoker or non-smoker.

Variables related to dental status: number of teeth present, total number of teeth with apical periodontitis, affected jaw, affected tooth/teeth, previous treatment (absent, endodontic treatment, composite filling), age of previous treatment (approximate age, in years, related by the patient), presence of fistula (yes/no), number of treatment sessions, initial PAI score, PAI score at 12 months, PAI score at 24 months.

Variables related to the general diseases: cardiovascular, respiratory, gastrointestinal (gastritis), hepatic, neuropsychiatric, blood, immune, allergic, and endocrine disorders.

For each patient, the following data were recorded by the treatment provider. All teeth were classified according to the Federation Dentaire International (FDI) nomenclature.

### 2.7. Statistical Analysis

Statistical analysis was performed by using IBM SPSS Statistics 25 and Microsoft Office Excel/Word 2021. Quantitative variables were tested for distribution using the Shapiro–Wilk test and were expressed as means with standard deviations or medians with interpercentile ranges. Independent quantitative variables with non-parametric distribution were tested using the Mann–Whitney U/Kruskal–Wallis H test, and any correlation between them was calculated using Spearman’s rho correlation coefficient. Quantitative related variables with a non-parametric distribution (PAI score) were tested between measurements using the Wilcoxon test.

The qualitative variables were expressed as absolute values or percentages. Differences between independent qualitative variables were tested using Fisher’s Exact tests. The Bonferroni-corrected Z-tests were used to refine the results obtained in the contingency tables. Comparison of the rate of healing between 12 months and 24 months was done using the McNemar Test.

Generalized estimating equations (GEE) with exchangeable correlation matrix structure and robust estimation were implemented to observe the effect of the analyzed variables on healing evolution and PAI score evolution. For quantitative variables, a linear distribution with the identity function was used for the models. For binomial qualitative variables, a binomial logistic distribution was used for the models. Overall effects were analyzed along with interaction terms with time for each variable. The threshold considered for the significance level for all tests was considered to be α = 0.05.

The study’s main objectives were to study associations between dental groups or types of dental treatments and other analyzed factors, mainly through contingency tables with Pearson Chi-Square Tests/Fisher’s Exact Tests. Sample size was calculated in GPower 3.1.9.7. Considering that the maximum degrees of freedom from an association of two nominal variables in a contingency table, each with four categories, have a value of 9, considering a medium effect size—w of 0.3, an alpha probability of 0.05, and a statistical power of 0.95, the minimal sample size to be tested was 263. Considering that the total sample size of analyzed teeth was 277, the validation of the tested assumption based on the proposed conditions can be considered.

## 3. Results

The study group consisted of 200 patients, who presented 277 teeth with apical periodontitis. The majority of individuals in this study were female (58.5%). The mean age was 36.57 ± 14.47 years, with a minimum of 15 years and a maximum of 72 years.

The mean number of teeth present was 23.73 ± 4.82, with a median of 26 teeth, and that of teeth with apical periodontitis was 1.93 ± 1.17, with a median of 2 teeth.

There were no significant differences between the sex of the patients and the total number of teeth with apical periodontitis (*p* = 0.739).

Among the patients included in the study, 34 (17%) were smokers.

The correlation between age and number of teeth present showed that patients of older age were significantly more frequently associated with a lower number of teeth present (*p* < 0.001, R = −0.626). The distribution of both variables was non-parametric according to the Shapiro–Wilk test (*p* < 0.001).

**Figure 1:**
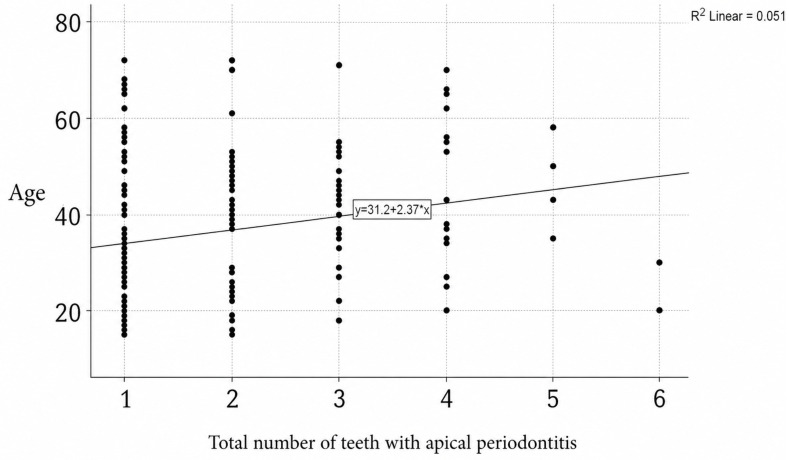
Correlation between age and the number of teeth with apical periodontitis.

In this treatment-based sample, there was a statistically significant positive association between age and the number of teeth with apical periodontitis (Spearman ρ = 0.275, *p* < 0.001), as shown in Figure 1 and Table 2. The distribution of both variables was non-parametric according to the Shapiro–Wilk test (*p* < 0.001).

Apical periodontitis had a significantly higher proportion in maxillary teeth (55.2%) compared to mandibular teeth (44.8%). The incisor group was the most affected (37.5%). The prevalence of periapical lesions in maxillary teeth was largest in maxillary lateral incisors (17.7%). In mandibular teeth, first molars had the largest prevalence of periapical lesions (16.2%). The most affected tooth was 22 (9%).

Of the total teeth analyzed, 62.8% had previous treatment, with root canal treatments being the most common in 39.7%. In 37.2% of cases, teeth with apical periodontitis presented carious processes.

The mean age of previous treatment was 3.06 ± 3.26 years, with a median of 3 years.

71.8% of the teeth were treated in a single session, and the main reason for presenting for treatment of the patients was pain (48.1%).

Fistula was observed in 16.6% of the patients.

The mean initial PAI score was 3.37 ± 0.9 points, with a median of 4 points; the final one was 1.19 ± 0.75 points, with a median of 1 point.

At the first recall (1 year), the teeth had healed in a proportion of 56.68%, and at the last recall, of 84.84%.

All dental characteristics of the studied group are presented in Table 3.

The data in Table 4 represent the distribution of the analyzed teeth by dental group and reason for presentation. The differences between groups are significant according to Fisher’s Exact Test (*p* < 0.001), and Z-tests with Bonferroni correction showed that incisors and canines were more frequently associated with a preprosthetic (esthetic) presentation, and premolars and molars were more frequently associated with a presentation for pain.

The data in Table 5 represent the distribution of the analyzed teeth according to dental group and prior treatment. As can be seen, incisors showed the most frequent previous endodontic treatment, and molars and premolars showed carious processes.

The differences between groups are significant according to Fisher’s Exact Test (*p* < 0.001) and Z-tests with Bonferroni correction show that teeth examined for pain were significantly more frequently associated with dental caries (22.38%), and teeth examined for periodic or preprosthetic control were significantly more frequently associated with root canal treatment (14.44%/15.16%).

Data from Table 6 and Table 7 represent the comparison of the age of previous dental treatment in relation to the dental group of the analyzed teeth. The age distribution was found to be non-parametric in most groups according to the Shapiro–Wilk test (*p* < 0.05). According to the Kruskal–Wallis H test, there are differences in age in relation to the dental group (*p* = 0.009) and post hoc tests show that incisors had a significantly higher age of previous treatment (median = 3 years, IQR = 0.25–5 years) compared to premolars (median = 1 year, IQR = 0–4 years) (*p* = 0.005).

The data in Table 8 and Table 9 represent the comparison of the age of previous dental treatment in relation to the prior treatment of the analyzed teeth. The age distribution is non-parametric in most groups according to the Shapiro–Wilk test (*p* < 0.05). According to the Kruskal–Wallis H test, there are differences in age in relation to the prior treatment (*p* < 0.001) and post hoc tests show that teeth with composite restoration (median = 4 years, IQR = 3–6 years) or root canal treatment (median = 4 years, IQR = 3–6 years) had a significantly higher age of previous treatment compared to teeth without prior treatment (median = 0 years, IQR = 0–0 years) (*p* < 0.001/*p* < 0.001) or compared to teeth with endodontic drainage (median = 0 years, IQR = 0–3 years) (*p* = 0.002/*p* = 0.001).

**Figure 2:**
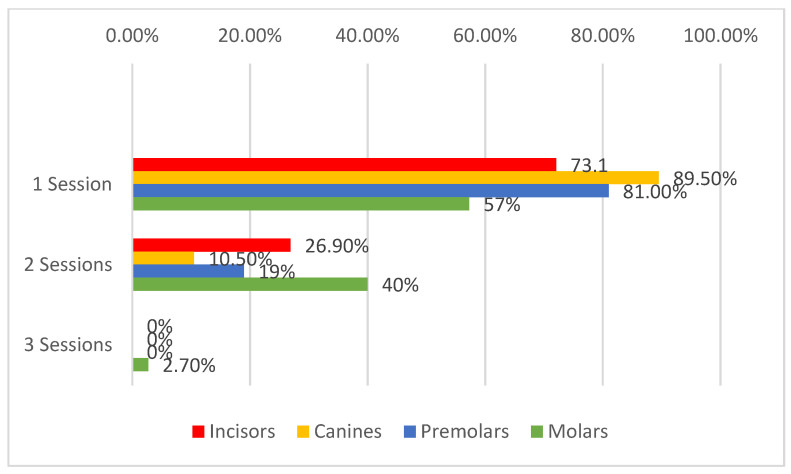
The distribution of analyzed teeth related to the number of treatment sessions and dental group.

The distribution of analyzed teeth related to the number of treatment sessions and dental group, as shown in Figure 2 and Table 10 revealed that the differences between groups are significant according to Fisher’s Exact Test (*p* = 0.007), and Z-tests with Bonferroni correction showed that the teeth that had one treatment session were significantly more frequently premolars than molars (81% vs. 57.3%) while teeth that had two treatment sessions were significantly more frequently molars than premolars (40% vs. 19%).

**Figure 3:**
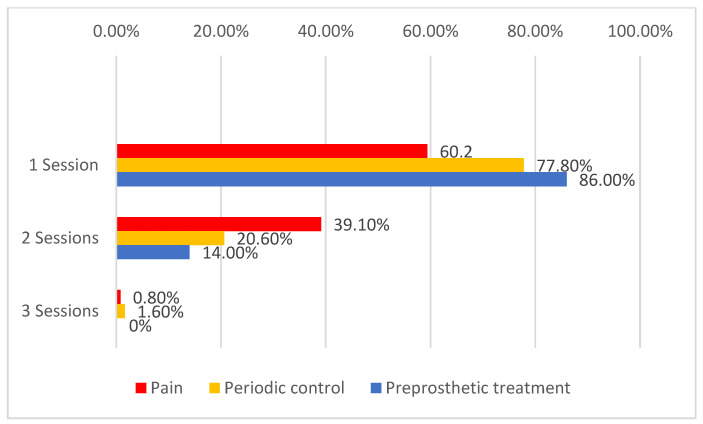
Distribution of analyzed teeth according to the number of treatment sessions and reasons for presentation.

The data in Figure 3 and Table 11 represent the distribution of analyzed teeth according to the number of treatment sessions and reason for presentation. Differences between groups are significant according to Fisher’s Exact Test (*p* < 0.001), and Z-tests with Bonferroni correction showed that teeth that had one treatment session were evaluated significantly more frequently with preprosthetic treatment or for periodic control than for pain (86%/77.8% vs. 60.2%). Teeth that had two treatment sessions were evaluated significantly more frequently for pain than preprosthetic or periodic control (39.1% vs. 14%/20.6%).

A detailed medical history was obtained for all patients at baseline. Overall, 16% of the study population presented at least one systemic condition. Cardiovascular diseases were the most frequent (8%), followed by allergic conditions (3%), neuropsychiatric disorders (2.5%), gastrointestinal disorders (2%), endocrine/metabolic disorders (2%), hepatic diseases (1.5%), immune-related disorders (1.5%), respiratory diseases (1%), and hematologic conditions (1%).

The relationship between 12-month healing status and the investigated factors was analyzed, and no statistically significant associations were identified (*p* > 0.05) (Table 12).

The relationship between healing status at 24 months and the investigated factors was analyzed, and no statistically significant associations were identified (*p* > 0.05) (Table 13).

**Figure 4:**
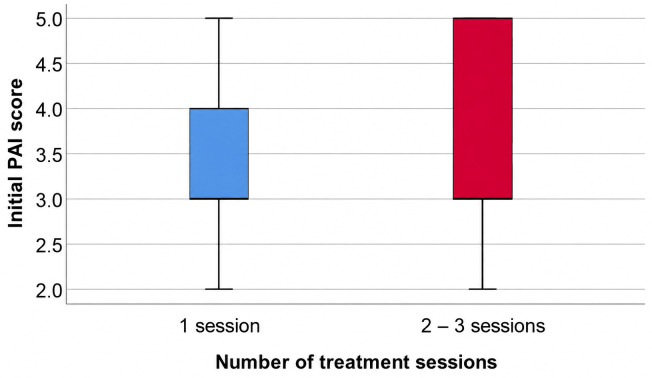
Comparison of the initial PAI score according to the number of treatment sessions.

Data from Figure 4 and Table 14 show the comparison of the initial PAI score according to the number of treatment sessions. Mean initial PAI score was 3.37 ± 0.9, median = 3 (IQR = 3–4). Distribution according to number of treatment sessions shows 200 teeth (72.2%) with one treatment session, 75 teeth (27.1%) with two treatment sessions and 2 teeth (0.7%) with three treatment sessions. Because of the small number of teeth in the last category, the last two categories were unified for a better comparison. According to the results, differences were significant (*p* = 0.016), teeth with two or three treatment sessions had significantly higher PAI values (median = 3, IQR = 3–5) in comparison to teeth with one treatment session (median = 3, IQR = 3–4).

**Figure 5:**
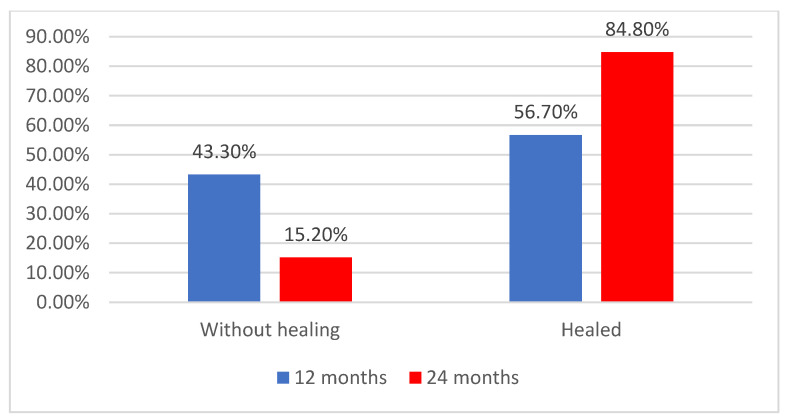
Comparison of healing frequency between 12 and 24 months.

**Figure 6:**
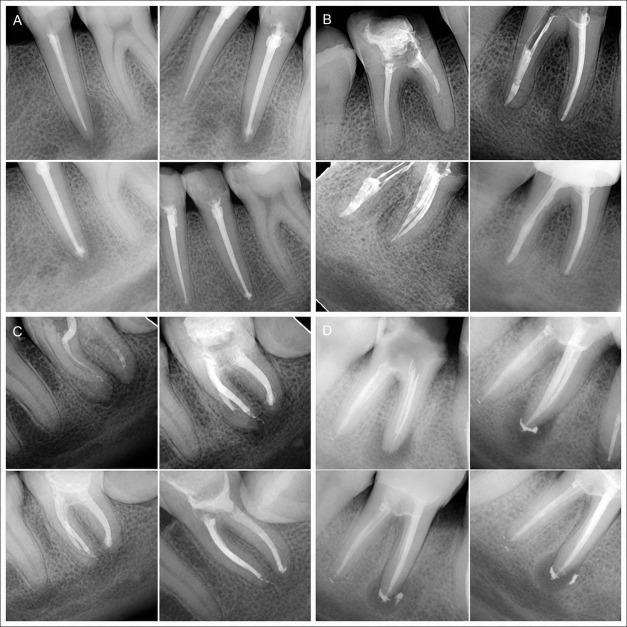
Postoperative and follow-up radiographs from representative cases with apical periodontitis. (**A**) Mandibular first premolar, 2-year follow-up, ranked as healed. (**B**) Mandibular first molar, 2-year follow-up, ranked as healed. (**C**) Mandibular second molar, 2-year follow-up. Apical extrusion of a separated instrument is present. The tooth met the predefined radiographic and clinical criteria for healing (PAI ≤ 2, asymptomatic), with no radiographic progression observed across follow-up; (**D**) Maxillary first premolar, 1-year follow-up, ranked as diseased.

**Figure 7:**
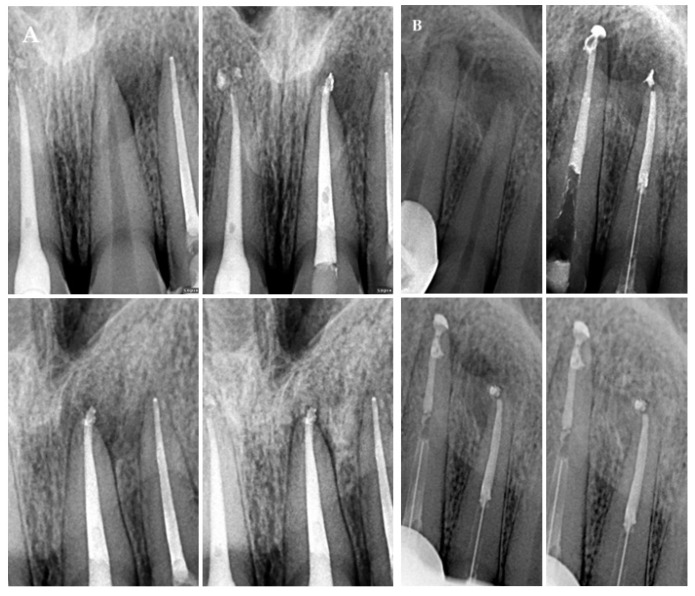
Postoperative and follow-up radiographs from representative cases with apical periodontitis. (**A**), First maxillary incisor, 2-year follow-up, ranked as healed. (**B**) Second maxillary incisor and canine, 2-year follow-up, ranked as healed.

The comparison of healing frequency between 12 and 24 months, Figure 5 and Table 15 showed that 42 teeth were non-healed at 12 months and remained non-healed at 24 months, 78 teeth were non-healed at 12 months and were healed at 24 months and 157 teeth were healed at 12 months and remained healed at 24 months. Differences between measurements show that the rate of healing significantly increased from 12 months to 24 months (84.8% vs. 56.7%) (*p* < 0.001). Postoperative and follow-up radiographs from representative cases with apical periodontitis are shown in Figure 6 and Figure 7.

The generalized estimating equations (GEE) models were used for the prediction of the influence of the analyzed factors on healing evolution. Results show that none of the analyzed variables had a significant effect on the overall healing rate (*p* > 0.05), and none of the interaction terms with time had a significant effect (*p* > 0.05). As such, the only model which is valid (M1.0) shows that the same result as in Table 15, as in, the healing rate at 24 months was significantly higher in comparison to the healing rate at 12 months by 4.277 times (95% C.I. = 3.174–5.761) (Beta = 1.453, 95% C.I. = 1.155–1.751) (*p* < 0.001) (Table 16).

The generalized estimating equations (GEE) models employed to predict the influence of analyzed factors on PAI score evolution revealed several significant findings (Table 17):

- Evolution in time of PAI score was significant (model M1.0) (Beta = −2.054, 95% C.I. = −2.181–−1.928) (*p* < 0.001), estimated marginal means show a significant decrease from the initial value (mean = 3.37, 95% C.I. = 3.27–3.48) versus the final value (mean = 1.32, 95% C.I. = 1.21–1.43);
- Age did not have a significant effect on overall PAI values (*p* = 0.477); however, age interaction over PAI evolution was significant (Beta = 0.009, 95% C.I. = 0.001–0.018) (*p* = 0.039), meaning that for each increase in one year of patients’ age, the decrease in PAI is lowered (as absolute value) by 0.009 units;
- Smoking (*p* = 0.231), Tooth type (*p* = 0.089), and Retreatment (*p* = 0.763) did not have a significant effect on overall PAI values, and interaction terms with time also did not have a significant effect;
- Teeth location had a significant effect on overall PAI values (Beta = 0.237, 95% C.I. = 0.060–0.414) (*p* = 0.009), showing that teeth on the mandible had significantly higher values than teeth on the maxilla;
- Comorbidities had no significant effect on overall PAI values (*p* = 0.998); however, the interaction term with time was significant (Beta = 0.410, 95% C.I. = 0.086–0.734) (*p* = 0.013), showing that patients with comorbidities had a significantly lower decrease in PAI by 0.410 units.

**Table 17:** Generalized estimating equations (GEE) models used for the prediction of analyzed factors influence on PAI score evolution.

Model	Variables	Wald X2 (Time)	*p*-Value (Tests of Model Effects)		
			Time	Predictor	Time × Predictor
M1.0	Time	1011.160	<0.001	-	-
M2.0	Time, Age	1011.160	<0.001	0.477	-
M2.1	Time, Age, Time × Age	175.942	<0.001	0.477	0.039
M3.0	Time, Smoking	1011.131	<0.001	0.231	-
M3.1	Time, Smoking, Time × Smoking	704.012	<0.001	0.231	0.141
M4.0	Time, Location	1011.160	<0.001	0.009	-
M4.1	Time, Location, Time × Location	968.336	<0.001	0.009	0.306
M5.0	Time, Tooth type	1011.160	<0.001	0.089	-
M5.1	Time, Tooth type, Time × Tooth type	722.089	<0.001	0.089	0.663
M6.0	Time, Comorbidities	1011.160	<0.001	0.998	-
M6.1	Time, Comorbidities, Time × Comorbidites	537.165	<0.001	0.998	0.013
M7.0	Time, Retreatment	1011.160	<0.001	0.763	-
M7.1	Time, Retreatment, Time × Retreatment	1005.302	<0.001	0.763	0.593

## 4. Discussion

Previous epidemiological studies obtained information about the periapical status from radiographic devices such as panoramic, periapical or CBCT [19,20,21,22]. In the present study, we used digital panoramic radiographs in conjunction with periapical radiographs of the affected tooth/teeth. Panoramic radiographs have a high specificity and sensitivity (86–96%) for the detection of periapical lesions, and are acceptable alternatives in studies on dental health [23]. The periapical radiographs provide improved visibility of dental structures and pathology, except for the maxillary second and third molars [24]. However, using only 2D radiography has limitations due to anatomical noise and geometric distortion, which can make detecting periapical lesions in cancellous bone less accurate [25,26,27]. CBCT is a more accurate diagnostic method, but because it is more precise and sensitive, it has the disadvantage of a higher level of ionizing radiation [28].

What is important to note in this study is that all patients who participated stated that during their previous dental treatments, the dentists did not use a dental dam, and the endodontic treatments were performed in several sessions. Thus, the teeth included in this study with previous endodontic treatments were deficient (>2 mm short of the radiographic apex and inadequate homogeneity). In order to develop ways to prevent tooth loss, it is important that doctors improve their techniques to avoid medical errors.

Similar to other studies, the sample in this study was 59% female and 41% male, which could be explained by the fact that women are more interested in their health or perhaps have a lower pain threshold than men [29,30]. Even though women were in the majority, there was no difference between the sexes regarding the total number of teeth with apical periodontitis. A study conducted in Brazil showed that the frequency of apical periodontitis is higher in women than in men [31], in contrast to a study conducted in Spain, where men were more affected [32]. Other studies from Greece and France found no correlation between sex and the prevalence of apical periodontitis [33,34,35].

The average number of teeth per person in this study was 23.73. This value is in agreement with other studies conducted on European populations. The average number of teeth with apical periodontitis was 2 [36,37]. In our study, there were no differences between the gender of the patients and the total number of teeth with apical periodontitis, thus confirming the results of other studies [33,38,39].

Regarding age and total number of teeth present, in our study, a significant decrease was observed in the age group of 30–39 years (24.05 ± 3.5, 25 (21–27)), a decrease observed to be slowed down towards 40–49 years/50–59 years/≥60 years. This can be explained by various factors such as economic status, level of education, demographic and socioeconomic factors. Age influenced the number of teeth with periodontitis, thus patients between 50 and 59 years were associated with a higher number (2.31 ± 1.28, 2 (1–3)), an aspect confirmed by other studies [32,40].

Regarding the location of apical periodontitis, it was more common in the maxilla than in the mandible (55.2% vs. 44.8%), similar to the results of previous studies [33,38]. This may be due to the fact that maxillary teeth are more susceptible to carious processes or the anatomical complexity they have, making endodontic treatments difficult to perform [41,42].

The results of our study showed that in the maxilla, the lateral incisors were the most affected, and in the mandible, the molar one. Incisors are most commonly affected due to dental trauma, which usually affects teeth that are located anteriorly [43]. In our study, teeth that presented trauma were excluded. On the other hand, the higher prevalence of first molars can be attributed to their shape, which includes several grooves that facilitate the accumulation of plaque. In addition, these teeth are usually the first permanent teeth to appear in the oral cavity [44]. Other factors that can favor the occurrence of caries and, respectively, the need for endodontic treatments due to their non-treatment are dental malposition, diet, and hygiene.

Etiology of apical periodontitis was associated more with endodontic treatment failure (39.7%), followed by dental caries (37.2%). Studies indicate a strong correlation between the quality of endodontic treatment and the presence of apical periodontitis [20,35]. It is well known that composite resins and adhesive systems can have a cytotoxic effect on the dental pulp [45,46]. However, another important factor affecting the vitality of the tooth is the rising intrapulpal temperature during preparation of the tooth and also residual dentin thickness [47,48,49]. In our study, 18.4% of the affected teeth presented composite restorations, but we do not know the technical details of how they were performed (we only know that the patients in this group had no previous dental dam treatments).

Clinically, the main reason for which patients presented to the clinic was pain (46.2%) caused by dental caries (22.38%), which is generally the most frequent reason for consultations in dentistry [50].

The large number of incisors present in our study may be due to the fact that patients are more interested in their appearance, as they want to have harmonious esthetics. Their esthetics can be affected by tooth discoloration caused by pulp necrosis, or by certain materials used in endodontic treatments [51].

In the present study, 72.2% of teeth were treated in one session and 27.1% in two sessions. According to some studies, there is no significant difference in the treatment outcomes between one-visit and two-visit endodontic treatments [52]. Calcium hydroxide dressing, standard material to use in root canal treatment between sessions, especially in very large periapical lesions is demonstrated to be useful. We used calcium hydroxide only when we could not obtain an optimal drying, due to persistent discharge from the root canal system [15,53,54].

A statistical difference was found between the size of the periapical lesion and the age of the patients. This correlation confirmed that advanced age can have a negative effect on the healing of periapical lesions. It is well known that healing is faster in young people compared to the elderly [55,56].

In our study, post-treatment evaluation was done at 12 months and it showed that 56.68% of the teeth were healed, 9.39% the lesion was unchanged, in 3.97% it was growing and 45.78% was decreasing. At 24 months, 84.84% of the teeth were healed, 1.44% the lesion was unchanged, in 5.05% it was growing and 8.66% was decreasing. Our findings are similar to other studies in which the success rate at the 2-year recall was noticeably higher than at the 1-year recall [57,58].

Placement of a crown restoration can improve periapical healing, and delayed placement of the final restoration might lead to failure, negatively affecting the long-term survival of the teeth [58,59]. These findings can limit the complications that can occur, like odontogenic infections of the face or cervical regions [60].

The present study did not reveal any statistically significant association between healing status at 12/24 months and the investigated variables, including systemic comorbidities, smoking status, age, retreatment, tooth type, and jaw location. This may suggest that, in the present cohort, these factors were not significant predictors of periapical healing after endodontic treatment. A possible explanation is that the healing outcome may depend more strongly on local treatment-related parameters, such as effective microbial control, adequate chemomechanical preparation, obturation quality, and coronal sealing, than on the patient- and tooth-related characteristics evaluated. However, this finding should be interpreted with caution, since the lack of statistical significance does not necessarily indicate the absence of a true association. The limited sample size, the small number of cases in certain subgroups, and the multifactorial nature of apical healing may have reduced the ability to detect subtle differences. Therefore, further large-scale prospective studies are required to better clarify the potential influence of these variables on endodontic treatment outcomes.

The present study demonstrates a statistically and clinically significant reduction in PAI scores over time (Beta = −2.054, 95% C.I. = −2.181 to −1.928, *p* < 0.001), with estimated marginal means decreasing from 3.37 (95% C.I. = 3.27–3.48) to 1.32 (95% C.I. = 1.21–1.43), providing robust evidence for the effectiveness of endodontic treatment in resolving periapical pathology. This substantial decrease in PAI scores aligns with the longitudinal study by Huumonen et al. [61], who documented similar PAI score reductions over 12-month follow-up periods. The systematic review and meta-analysis by Ng et al. [62] corroborates this finding with a weighted pooled success proportion of 81% across 24 studies.

The age-time interaction (β = 0.009, *p* = 0.039) indicates that younger patients experienced more pronounced PAI score reductions, suggesting age-related differences in periapical healing capacity. This finding corroborates the work Trusewicz et al. [63] who similarly reported superior healing outcomes in younger patients. Conversely, Marquis et al. [64] found no significant age effect. From a clinical perspective, this emphasizes the importance of considering patient age when establishing treatment prognoses and may justify more conservative observation periods in younger populations.

The anatomical location of treated teeth emerged as a significant predictor of overall PAI values, with mandibular teeth exhibiting higher scores compared to maxillary teeth. This difference may be attributed to the greater density of mandibular cortical bone, which could impede the rate of periapical healing and affect the radiographic presentation of lesions. Additionally, the thicker cortical plates in the mandible may result in more pronounced radiographic appearance of periapical pathology, potentially inflating PAI scores independent of actual lesion severity—a phenomenon documented by Bender and Seltzer [65] in their classic radiographic study.

A particularly noteworthy finding was the significant interaction between comorbidities and time (β = 0.410, *p* = 0.013), revealing that patients with systemic health conditions experienced attenuated PAI score reduction compared to systemically healthy individuals. This observation supports the findings of Fouad and Burleson [66] who demonstrated that patients with diabetes showed significantly delayed periapical healing, and aligns with the meta-analysis by Segura-Egea et al. [67] which established clear associations between systemic diseases and endodontic outcomes. Specifically, diabetic patients in the study by Arya et al. [68] showed lower healing rates. This observation has profound clinical implications, as it suggests that comorbidities may compromise the periapical healing process through mechanisms including impaired microcirculation, altered immune function, and disrupted bone remodeling. The magnitude of this effect (0.410 units) represents a clinically meaningful reduction in healing rate, suggesting that patients with comorbidities may require an extended follow-up period. This finding reinforces the importance of comprehensive medical history evaluation and interdisciplinary communication in endodontic treatment planning, particularly for medically complex patients.

Interestingly, several factors hypothesized to influence PAI scores did not demonstrate significant effects in our model. Smoking status, despite its well-documented negative impact on wound healing and bone metabolism in other oral surgical contexts, did not significantly affect PAI evolution (*p* = 0.231). This unexpected finding contradicts several previous studies, including Paljevic et al. [69] who reported a lower success rate in smokers, and Majid OW [70] who documented delayed healing in smoking patients. This unexpected finding may reflect several possibilities: our sample size may have been insufficient to detect a modest smoking effect; the intensity and duration of smoking (pack-years) may be more critical than binary smoking status or contemporary endodontic techniques may effectively overcome smoking-related healing impairments. Similarly, tooth type (*p* = 0.089) and retreatment status (*p* = 0.763) did not significantly influence outcomes. Regarding retreatment, our findings align with Moazami et al. [71], who reported comparable success rates between primary treatment and retreatment when proper protocols were followed, but contrast with the study by Artaza et al. [72] which reported that retreatment exhibited lower overall success compared with initial treatment under the loose criterion (79% vs. 89%). These findings suggest that when standardized clinical protocols and adequate operator expertise are applied, the fundamental biological healing response transcends anatomical tooth variations and prior treatment history.

A key limitation of this study lies in our primary reliance on the Periapical Index (PAI) as the sole measure of periapical healing. While a reduction in the PAI score provides valuable evidence of radiographic improvement and healing progress, it is critical to understand that this does not automatically equate to complete, long-term biological resolution. A true determination of biological healing requires a more comprehensive assessment, and future studies would ideally incorporate other diagnostic tools, such as targeted microbiological or molecular analyses, or three-dimensional imaging (CBCT), where ethically and practically feasible. These complementary methods could provide a more in-depth understanding of the biological changes occurring in the periapical tissues. Nonetheless, the PAI remains a well-established and standardized outcome measure in endodontic research, and its use in this study allowed for a rigorous and comparable evaluation of treatment outcomes within our specific cohort.

The study sample was selected from consecutive patients presenting for endodontic treatment at the study host clinic, and therefore, the lower proportion of maxillary and molar teeth was not due to intentional sampling bias. However, the uneven distribution of tooth types remains an important limitation. Since AP is commonly observed in molars, particularly maxillary first molars, the underrepresentation of these teeth may affect the generalizability of the findings.

A limitation of this study is its retrospective design and the inclusion of multiple teeth from single individuals. Among the 277 teeth analyzed, several came from the same patient. This introduces a ‘clustering’ effect, meaning that observations for these teeth are not completely independent, as they share patient-specific systemic and biological factors. While teeth were analyzed as independent units, this lack of independence could affect the precision of our statistical estimates. Future studies should ideally use multilevel statistical models, such as mixed-effects models, to better account for this clustering.

A further limitation concerns the interpretation of cases affected by procedural complications. In the present cohort, one tooth presented apical extrusion of a separated instrument. Although this tooth met the predefined radiographic and clinical criteria for healing (PAI ≤ 2, absence of symptoms) and showed no radiographic progression over the 24-month follow-up, instrument separation with apical extrusion is an undesirable procedural complication, and the long-term behavior of an extruded fragment cannot be fully predicted from two-dimensional radiography and symptom assessment alone.

Apical periodontitis is a clinically important condition that may compromise tooth survival if not adequately treated. The higher frequency observed in elderly patients may reflect cumulative dental disease and previous restorative or endodontic interventions. The predominance of maxillary incisors may also reflect patient-related treatment-seeking behavior, as anterior teeth have greater esthetic importance and may prompt patients to seek dental care more readily than posterior teeth. The increased healing rates from 12 to 24 months confirm that periapical repair is time-dependent and highlight the importance of long-term follow-up. To the best of our knowledge, this is the first study from this region of Romania to assess the distribution and radiographic healing of apical periodontitis after endodontic treatment and retreatment. However, the results should be interpreted cautiously because healing was assessed mainly by the PAI score. Although a limited amount of data was obtained, it should be enough to form an overview of this pathology in this region.

## 5. Conclusions

The findings from this research reveal a significant occurrence of apical periodontitis resulting from substandard root canal procedures. Female patients demonstrate a markedly higher tendency to seek dental care compared to their male counterparts. There were no statistically significant associations between healing status at 12 and 24 months and the investigated variables, including systemic comorbidities, smoking status, age, retreatment, tooth type, and jaw location. Considerable initiatives are required to enhance the quality of endodontic treatment practices. Implementing well-designed endodontic protocols can consistently achieve favorable outcomes with high success rates.

## Figures and Tables

**Table 1:** Diagnostic criteria for different types of periodontitis.

Periodontitis	Clinical Signs	Radiological Signs
Symptomatic Apical Periodontitis	Spontaneous Pain +++ Pain to percussion +++ Lack of pulpal sensitivity +++ Pain on palpation apical +++	Thickening periodontal ligament +++ Radiolucency, image of bone lysis +
Chronic Apical Abscess	Presence of fistula +++ Lack of sensitivity therefore +++ Pain to apical palpation ++	Radiolucency, image of bone lysis +++ Thickening periodontal ligament +++
Asymptomatic apical periodontitis	Lack of sensitivity therefore +++	Radiolucency, image of bone lysis +++
Acute Apical Abscess	Swelling +++ Pain to percussion + Lack of pulpal sensitivity +++ Pain to apical palpation	Radiolucency, image of bone lysis +++ Thickening periodontal ligament

+++, high intensity; ++, moderate intensity; +, low intensity

**Table 2:** Correlation between age and the number of teeth with apical periodontitis.

Correlation	*p* *
Age (*p* < 0.001 **) × Total number of teeth with apical periodontitis (*p* < 0.001 **)	<0.001, R = 0.275

* Spearman’s rho Correlation Coefficient, ** Shapiro–Wilk Test.

**Table 3:** Dental characteristics of the studied group.

Parameter	Value
Localization	153 (55.2%) maxillary 124 (44.8%) mandible
Dental group	104 (37.5%) Incisive 19 (6.9%) Canine 79 (28.5%) Premolar 75 (27.1%) Molar
Tooth affected	41 (14.8%) Maxillary central incisors 49 (17.7%) Maxillary lateral incisors 11 (4%) Maxillary canines 26 (9.4%) Maxillary premolars 1 20 (7.2%) Maxillary premolars 2 9 (3.2%) Maxillary molars 1 4 (1.4%) Maxillary molars 2 5 (1.8%) Mandibular central incisors 9 (3.2%) Mandibular lateral incisors 9 (3.2%) Mandibular canines 11 (4%) Mandibular premolars 1 21 (7.6%) Mandibular premolars 2 45 (16.2%) Mandibular molars 1 17 (6.1%) Mandibular molars 2
Appointment reason	128 (46.2%) Pain, 63 (22.7%) Periodic control 86 (31%) Preprosthetic
Previous treatment duration (years) (Mean ± SD, Median (IQR))	3.06 ± 3.26, 3 (0–5)
Baseline PAI Score (Mean ± SD, Median (IQR)	3.37 ± 0.9, 3 (3–4)
Final PAI Score (Mean ± SD, Median (IQR)	1.31 ± 0.93, 1 (1–1)
Evolution at the first recall	157 (56.68%) Healed 26 (9.39%) Stationary 11 (3.97%) Failure 83 (45.78%) Healing
Evolution at the last recall	235 (84.84%) Healed 4 (1.44%) Stationary 14 (5.05%) Failure 24 (8.66%) Healing

**Table 4:** Distribution of analyzed teeth related to dental group and reason for presentation.

Dental Group /Appointment Reason	Pain		Periodic Recall		Preprosthetic		*p*
	No.	%	No.	%	No.	%	
Incisives	32	25%	22	34.9%	50	58.1%	<0.001
Canines	3	2.3%	5	7.9%	11	12.8%	
Premolars	46	35.9%	16	25.4%	17	19.8%	
Molars	47	36.7%	20	31.7%	8	9.3%	

**Table 5:** Distribution of analyzed teeth related to the number of treatment sessions and prior treatment.

Dental Group	Tooth Decay		Dental Filling		Endodontic Drainage		Endodontic Treatment	
Prior Treatment	No.	%	No.	%	No.	%	No.	%
Incisives	28	26.92%	28	26.92%	4	3.85%	44	42.31%
Canines	9	47.37%	1	5.26%	0	0.00%	9	47.37%
Premolars	34	43.04%	10	12.66%	5	6.33%	30	37.97%
Molars	32	42.67%	12	16.00%	4	5.33%	27	36.00%

**Table 6:** Comparison of the age of previous dental treatment in relation to the dental group of the analyzed teeth.

Dental Group	Mean ± SD	Median (IQR)	Mean Rank	*p* *
Incisors (*p* < 0.001 **)	3.54 ± 3.09	3 (0.25–5)	154.62	0.009
Canines (*p* = 0.001 **)	4.74 ± 5.56	3 (0–10)	150.47	
Premolars (*p* < 0.001 **)	2.06 ± 2.53	1 (0–4)	115.77	
Molars (*p* < 0.001 **)	3.01 ± 3.16	3 (0–5)	138.90	

* Kruskal–Wallis H test, ** Shapiro–Wilk test.

**Table 7:** Comparison of the age of previous dental treatment in relation to the dental group of the analyzed teeth.

Dental Group *	Incisors	Canines	Premolars	Molars
Incisors	-	1.000	0.005	1.000
Canines	1.000	-	0.490	1.000
Premolars	0.005	0.490	-	0.396
Molars	1.000	1.000	0.396	-

* Dunn–Bonferroni post hoc test.

**Table 8:** Comparison of the age of previous dental treatment in relation to the previous treatment.

Previous Treatment	Mean ± SD	Median (IQR)	Mean Rank	*p* *
Absent (*p* < 0.001 **)	0.89 ± 2.58	0 (0–0)	73.63	<0.001
Composite restoration (*p* = 0.099 **)	4.33 ± 2.35	4 (3–6)	104.09	
Endodontic drainage (*p* < 0.001 **)	1.69 ± 3.06	0 (0–3)	97.50	
Previous endodontic treatment (*p* < 0.001 **)	4.65 ± 3.02	4 (3–6)	184.21	

* Kruskal–Wallis H Test, ** Shapiro–Wilk Test.

**Table 9:** Comparison of the age of previous dental treatment in relation to the previous treatment.

Previous Treatment *	Absent	Composite Restoration	Endodontic Drainage	Previous Endodontic Treatment (*p* < 0.001 *)
Absent	-	<0.001	1.000	<0.001
Composite restoration	<0.001	-	0.002	1.000
Endodontic drainage	1.000	0.002	-	0.001
Previous endodontic treatment (*p* < 0.001 *)	<0.001	1.000	0.001	-

* Dunn–Bonferroni Post Hoc Test.

**Table 10:** The distribution of analyzed teeth related to the number of treatment sessions and dental group.

Nr. Sessions /Dental Group	Incisors		Canines		Premolars		Molars		
	Nr.	%	Nr.	%	Nr.	%	Nr.	%	
1 Session	76	73.1%	17	89.5%	64	81%	43	57.3%	*p* *
2 Sessions	28	26.9%	2	10.5%	15	19%	30	40%	
3 Sessions	0	0%	0	0%	0	0%	2	2.7%	

* Fisher’s Exact Test.

**Table 11:** Distribution of analyzed teeth according to the number of treatment sessions and reasons for presentation.

Nr. of Treatment Sessions /Reason for Presentation	Pain		Periodic Control		Pain		
	Nr.	%	Nr.	%	Nr.	%	
1 Session	77	60.2%	49	77.8%	74	86%	*p* *
2 Sessions	50	39.1%	13	20.6%	12	14%	
3 Sessions	1	0.8%	1	1.6%	0	0%	

* Fisher’s Exact Test.

**Table 12:** Distribution of the patients according to the existence of healing at 12 months and other analyzed factors.

Healing/ Comorbidities	Absent (*N* = 120)		Present (*N* = 157)		*p* *
	Nr.	%	Nr.	%	
Comorbidities	17	14.2%	28	17.8%	0.511
Cardiovascular	11	9.2%	15	9.6%	1.000
Respiratory	0	0%	3	1.9%	0.261
Gastrointestinal	0	0%	4	2.5%	0.136
Hepatic	2	1.7%	1	0.6%	0.581
Neuropsychiatric	3	2.5%	2	1.3%	0.655
Hematologic	2	1.7%	1	0.6%	0.581
Autoimmune	1	0.8%	3	1.9%	0.637
Alergic diseases	2	1.7%	6	3.8%	0.473
Endocrine	3	2.5%	5	3.2%	1.000
Smoking	20	16.7%	32	20.5%	0.442
Age (Median (IQR))	33.5 (24.25–50)		35 (25–47.5)		0.545 **
Retreatment (Nr., %)	54	45%	69	43.95%	0.717
Teeth type (Nr. %)					
Incisors	43	35.8%	61	38.9%	0.521
Canines	6	5%	13	8.3%	
Premolars	34	28.3%	45	28.7%	
Molars	37	30.8%	38	24.2%	
Location (Nr., %)					
Maxilla	63	52.5%	90	57.3%	0.465
Mandible	57	47.5%	67	42.7%	

* Fisher’s Exact Test, ** Mann–Whitney U Test.

**Table 13:** Distribution of the patients according to the existence of healing at 24 months and other analyzed factors.

Healing/ Comorbidities	Absent (*N* = 42)		Present (*N* = 235)		*p* *
	Nr.	%	Nr.	%	
Comorbidities	8	19%	37	15.7%	0.649
Cardiovascular	4	9.5%	22	9.4%	1.000
Respiratory	0	0%	3	1.3%	1.000
Gastrointestinal	0	0%	4	1.7%	1.000
Hepatic	1	2.4%	2	0.9%	0.391
Neuropsychiatric	2	4.8%	3	1.3%	0.166
Hematologic	2	4.8%	1	0.4%	0.061
Autoimmune	1	2.4%	3	1.3%	0.476
Alergic diseases	0	0%	8	3.4%	0.612
Endocrine	1	2.4%	7	3%	1.000
Smoking	8	19%	44	18.8%	1.000
Age (Median (IQR))	36 (23.75–47)		34 (25–49)		0.935 **
Retreatment (Nr., %)	23	54.8%	100	42.55%	0.739
Teeth type (Nr. %)					
Incisors	14	33.3%	90	38.3%	0.347
Canines	3	7.1%	16	6.8%	
Premolars	9	21.4%	70	29.8%	
Molars	16	38.1%	59	25.1%	
Location (Nr., %)					
Maxilla	20	47.6%	133	56.6%	0.314
Mandible	22	52.4%	102	43.4%	

* Fisher’s Exact Test, ** Mann–Whitney U Test.

**Table 14:** Comparison of the initial PAI score according to the number of treatment sessions.

Treatment Sessions	Mean ± SD	Median (IQR)	Mean Rank	*p* *
1 treatment session	3.28 ± 0.84	3 (3–4)	132.23	0.016
2–3 treatment sessions	3.61 ± 1.01	3 (3–5)	156.58	

* Mann–Whitney U Test.

**Table 15:** Comparison of healing frequency between 12 and 24 months.

Healing	12 Months	24 Months	*p* *
Nr.	157	235	<0.001
%	56.7%	84.8%	

* Related-Samples McNemar Test.

**Table 16:** Generalized estimating equations (GEE) models used for the prediction of analyzed factors influence over healing evolution.

Model	Variables	Wald X2 (Time)	*p*-Value (Tests of Model Effects)		
			Time	Predictor	Time × Predictor
M1.0	Time	91.327	<0.001	-	-
M2.0	Time, Age	91.238	<0.001	0.783	-
M2.1	Time, Age, Time × Age	14.222	<0.001	0.900	0.716
M3.0	Time, Smoking	91.044	<0.001	0.513	-
M3.1	Time, Smoking, Time × Smoking	53.654	<0.001	0.713	0.469
M4.0	Time, Location	91.815	<0.001	0.327	-
M4.1	Time, Location, Time × Location	91.015	<0.001	0.268	0.587
M5.0	Time, Tooth type	91.898	<0.001	0.433	-
M5.1	Time, Tooth type, Time × Tooth type	61.271	<0.001	0.375	0.448
M6.0	Time, Comorbidities	90.771	<0.001	0.605	-
M6.1	Time, Comorbidities, Time × Comorbidites	49.764	<0.001	0.949	0.166
M7.0	Time, Retreatment	66.959	<0.001	0.661	-
M7.1	Time, Retreatment, Time × Retreatment	66.598	<0.001	0.651	0.906

## Data Availability

The original contributions presented in this study are included in the article. Further inquiries can be directed to the corresponding authors.
